# Self-reported life experiences of members of the LGBTQ+ community in Accra, Ghana

**DOI:** 10.1371/journal.pone.0325148

**Published:** 2025-05-29

**Authors:** Donald Womonia Anuga, Merri Iddrisu, Kennedy Dodam Konlan

**Affiliations:** Department of Adult Health, School of Nursing and Midwifery, University of Ghana, Legon, Greater Accra Region, Ghana; Australian Catholic University, AUSTRALIA

## Abstract

**Background:**

Lesbian, Gay, Bisexual, Transgender, Queer/Questioning (LGBTQ+) community members encompass individuals whose sexual orientations and gender identities do not align with the heterosexual norms in society. Even though this population has gained increasing prominence worldwide, most societies particularly in sub-Saharan Africa (SSA) abhor these sexual orientations. We explored the self-reported life experiences of members of the LGBTQ+ community in Accra, Ghana.

**Methods:**

In this qualitative enquiry, we recruited fifteen (15) members of the LGBTQ+ community in Accra via purposive snowballing sampling technique. We conducted in-depth interviews using a pre-tested interview guide and the interviews were audio taped and transcribed verbatim. Thematic analysis was conducted with the aid of NVivo 10.0.

**Results:**

The participants recounted their experiences within their individual families, within the LGBTQ+ community, and their experiences with the public in Ghana and as well as within their extended families. The participants claimed that the general society (public) in Ghana stigmatizes against members of the LGBTQ+ community with most families rejecting members who claim to be LGBTQ+ persons. The participants however claimed that the LGBTQ+ community served as a major source of support for them and helped them cope with the rejection and stigma associated with their sexual orientations. They further claimed that the stigma and rejection contributed to poor mental health of most LGBTQ+ persons with most of them seeking asylum in more inclusive and welcoming jurisdictions.

**Conclusion:**

Members of the LGBTQ+ community in Ghana face stigma and societal pressures in their families and public spaces, creating stress that contributes to mental health challenges. We recommend that the Government of Ghana through the Ministry of Gender, Children and Social Protection and the Ghana Health Service increases public education to improve cultural competence and inclusivity of this vulnerable population in society to reduce stigma and discrimination.

## Introduction

Individuals who identify as Lesbian, Gay, Bisexual, Transgender, Queer/Questioning (LGBTQ) persons globally are said to experience harsh discrimination and inequalities both in public and private life [1–8]. These experiences are even more aggravated in countries who do not accept these specific sexual orientations [2,6]. Worldwide, efforts are being put in place to improve the life experiences [6,7] and quality of life of these LGBTQ+ community members [1–5]. Even though accurate global statistics on the experiences of members of the LGBTQ community are challenging to obtain due to various factors, including underreporting [2,3]. This situation is more pronounced in developing countries like those in Africa and Asia because same-sex sexual activity is still criminalized in more than seventy (70) countries with majority of such countries in Asia and Africa [1,4].

In the African context, obtaining precise data on the number of LGBTQ+ persons and their lived experiences are challenging due to under reporting occasioned by cultural and legal constraints that abhor LGBTQ+ [1,2]. Several African nations still have laws and policies that negatively affect LGBTQ individuals [6]. For instance, Men who engage in homosexual behavior risk the death sentence in Sudan, Mauritania, Somalia, and some regions of northern Nigeria; life in prison in Uganda, Tanzania, and Sierra Leone; and lengthy prison terms in Kenya, Malawi, Senegal, and the Gambia [6]. In Nigeria, heterosexual friends, family members, and allies who assist or support gay and lesbian men and women run the possibility of receiving a ten-year prison term [7]. In South Africa and Cape Verde, liberal attitudes and strong constitutional protections for sexual minority rights have fallen short of properly defending LGBTQ+ communities from prejudice, stigma, and violence even though LGBTQ is legalized in these nations [8]. Lesbian, gay, bisexual, transgender and queer/questioning (LGBTQ) is largely considered to be an immoral act in Africa and is criminalized by most national laws [1] and this makes it difficult to explore the lived experiences of this unique population. Nonetheless, the LGBTQ+ community is expanding in many African countries such as South Africa, Kenya and Tunisia [2] with anecdotal and empirical evidence [1–7] of abuse by heterosexual counterparts.

In Ghana, lesbian, gay, bisexual, transgender and queer/questioning (LGBTQ) has gained national level discussion over the years where many prominent academics and social advocates have openly supported its decriminalization and acceptance [9,10]. Despite the growing discussion on decriminalizing LGBTQ+ activity in Ghana, there is a strong disapproval of its acceptance from many quarters such as the religious societies, civil society groups and ordinary citizens [9,10]. Indeed, Ghana‘s 8^th^ parliament had passed a law that was awaiting presidential assent and if it had been assented to, will further criminalize the activities of LGBTQ+ in the country and this has pushed this key population into secrecy [10]. The issue of further legislating to ban the practice of LGBTQ+ in Ghana is still being discussed by the 9^th^ parliament of Ghana and this could further negate the gains made in protecting the rights of these minority populations.

Even though the LGBTQ+ community has gained increasing visibility and recognition worldwide since the 1990s [8,10], this progress has not translated into improved quality of life for LGBTQ+ individuals, who are said to experience disparities in almost all facets of life compared to their heterosexual counterparts [5,8]. Across Africa, LGBTQ practices are widely stigmatized and criminalized with most persons identifying as LGBTQ+ shunned by the general society [10]. A similar narrative exists in Ghana where religious and culturally opinions do not permit members of the LGBTQ+ community to live a free life in society [9,11] with anecdotal evidence suggesting attacks on members of the LGBTQ+ community. Traditional norms and religious practices abhor LGBTQ+ practices in Ghana and often fuel stigma, discrimination and sometimes persecution of LGBTQ+ persons [11]. These could predispose LGBTQ+ persons in Ghana to social isolation and poor quality of mental health of these unique individuals. There is however paucity of data on the lived experiences of LGBTQ+ persons in the Ghanaian society.

### Aim

We explored the self-reported life experiences of members of the LGBTQ+ community in Accra, Ghana.

## Methods

### Research design

The study used a qualitative exploratory descriptive design to explore the self-reported life experiences of members of the LGBTQ+ in Ghana.

### Study setting

This study was conducted in the Accra Metropolis, which is the largest urban town in Ghana in relation to population and is the commercial and political capital of the country [11]. The urban diversity of the Accra metropolis influenced its selection as the study setting. Moreover, the decision was also substantiated by a nationwide behavioral epidemiological survey targeting men who have sex with men (MSM) in Ghana where it was revealed that a considerable number of LGBTQ+ live in Accra [7]. Even though majority of the residents of Accra are heterosexual, there are reports of some LGBTQ+ persons living in Accra [7,11] with most of them reportedly living in fear due to their purported persecution owning to their sexual orientations and preferences.

The researchers herein were introduced to a clinic providing health care for the special needs of the LGBTQ+ community in the Eastern part of Accra, Ghana by a colleague professional nurse during a round table discussion on HealthCare Access for Vulnerable Populations at the Scientific Conference of the College of Health Sciences, University of Ghana organized in Accra, Ghana in 2023. The unique challenges identified during the round-table discussions at the conference served as an impetus for the conduct of the study. The researchers, after obtaining ethical approval for the study, were led to the said clinic in the Eastern part of Accra for community entry by a nurse manager in the said facility. The necessary community entry protocols were undertaken after the patients and managers of the facility had been assured of strict adherence to the ethical principles of research; specifically, privacy, anonymity, confidentiality among others.

### Population, sampling, and sample size

The LGBTQ community members in the Accra Metropolis who self-identified as members of the LGBTQ+ community for the past one year formed the study population.

Purposive snowball sampling technique was adopted for this study [12]. The first participant was identified in the special clinic located at the Eastern part of Accra providing services for LGBTQ+ persons. The said participant was receiving treatment for a complication due to his sexual orientation as an LGBTQ+ person and consented to be recruited for the study for the improvement of the quality of life of LGBTQ+ persons in Ghana. The participant was willing to take part in the study as long as the researchers would adhere to confidentiality and would not disclose the specific location of the said clinic in Accra to the public as he feared the clinic could be attacked by the public. This first contact person who consented to be part of the study then introduced other members of the LGBTQ+ community who were subsequently recruited after they had equally consented to be part of the study. Participants who declined consent were not recruited.

The sample size for the study was based on the principle of data saturation [11,12] and this was achieved at the conclusion of the fifteenth participant interview.

### Inclusion criteria

The inclusion criteria were self-identified members of the LGBTQ+ community for the past one year in Ghana who were the age of majority (Eighteen years)

### Exclusion criteria

The study excluded minors and individuals who were less than one year as members of the LGBTQ+ community in Ghana. Also members of the LGBTQ+ community who were critically ill were excluded.

### Data collection and tools

Data collection was done at one of the consulting rooms of the special clinic located at the Eastern part of Accra which had been assigned to the researchers by the nurse manager of the facility post the completion of community entry protocol at the clinic. The researchers presented the participant information sheets, informed consent forms and tools for the data collection to the prospective participants and explained the rationale for the study to them. The potential participants were allowed to ask questions and feedback provided. Also, issues of anonymity, privacy, and confidentiality among others were emphasized during the interaction with the potential participants at the out-patient Department of the clinic. We encouraged the participants to enroll unto the study for the advancement of their quality of life based on the findings of the study and encouraged them to recommend other members of the LGBTQ+ community to take part in the study.

Even though the interviews were supposed to be conducted in English and any of the local languages within the Accra metropolis depending on the participant ‘s language preference, all the participants spoke English during the interviews.

Data collection took place over a two-month period, from April to May, 2024.

A pre-tested semi-structured interview guide (S1) was developed, and it had two parts; Section A and Section B. Section A captured the participants’ demographic data, including age, sex, educational level, occupation, gender identity, and sexual orientation. Section B comprised open-ended questions, supplemented with probes where necessary, which explored self-reported life experiences of members of the LGBTQ+ community in Accra, Ghana.

Each participant’s interview lasted between 35–45 minutes.

The interviews were conducted at one of the consulting rooms at the clinic where the LGBTQ+ persons were receiving care. This clinic was seen as a haven for members of the LGBTQ+ persons as it was a usual place that they came to for healthcare. The consulting room at the clinic served as a natural environment where the LGBTQ+ persons could express themselves without fear since they have often visited the said facility. We encouraged the participants to recommend other LGBTQ+ persons to visit the clinic and take part in the study to help in the advancement of science and possibly improve their quality of life.

Throughout the course of gathering data, the researcher maintained a field journal. The diary entries were organized in a chronological order based on the time and date of each interview. Participants’ non-verbal clues, such as facial expressions, were recorded in the field diary. Additionally, we documented any other events that occurred during data collection that influenced the collected information.

The data saturation in this research was achieved after the 15^th^ participant’s interview.

### Data quality control

In this study we maintained the quality of the data collected by ensuring that the instrument for data collection was pre-tested among a similar population (LGBTQ+ persons) in the second largest city in Ghana (Kumasi). The individuals for pretesting of the study tool were selected from a clinic providing services for LGBTQ+ individuals in the Greater Kumasi Metropolis. This process was useful in refining the questions to suit the objectives of the study. We further broadened the scope of the questions to collect in-depth information to fit the methodology adopted for the study. Throughout the conception of the study, data collection and analysis of the data, we adhered to the methodological rigor required of qualitative studies. We ensured that the participants reviewed the transcripts to be sure they reflected their views from the audio interviews. The participants confirmed the word transcripts as verbatim transcriptions of their audio interviews.

### Methodological Rigour

We pre-tested the interview guide (S1) among a similar population (among two individuals who identified as being members of the LGBTQ+ community) in the second largest city in Ghana (Kumasi Metropolis). This exercise helped to modify the interview guide to cover every aspect of the experiences of the participants in line with the objectives of the study. Additionally, a purposive snowballing sampling technique was employed to ensure only individuals who identified as part of the LGBTQ+ community for more than one year who could give a vivid account of their life experiences as members of the LGBTQ+ were recruited. We used probing questions to elicit detailed information on the study objective. In addition, we have thoroughly described the data collection procedure in the methods to ensure transferability. Also, the data collection tool was semi-structured to ensure consistency in the questioning during the in-depth interviews. We further maintained a field notebook which documented important information and special events during the data collection as well as observed cues and facial expressions. Since the researchers were heterosexual persons, we bracketed our experiences and presuppositions during the data collection, analysis and writing of the manuscript. The researchers who were heterosexual individuals consistently ensured that their preconceptions of LGBTQ + , their personal bias towards LGBTQ+ individuals, and their assumptions did not influence the data collection, analysis and interpretation of the data.

### Reflexivity

The researchers maintained an ethical and professional outlook throughout the study. We did not allow our sexual preferences of being heterosexual to influence the perceptions we had about the study participants. As faculty members of the University of Ghana, we maintained our outmost fidelity to the advancement of science and gave a vivid account of the study findings without letting our personal biases to influence the study findings.

### Data analysis

The data analysis was done concurrently with data collection with the aid of Nvivo 10.0. The researchers adopted the thematic analysis approach to data analysis as recommended in literature [12]. The interviews that were audio recorded were transcribed word for word as soon as they were concluded. The audio transcripts in English were confirmed as verbatim transcripts of the audio files. The verbatim transcripts and the field notes recorded in the diary were used in the thematic analysis. We read, re-read, organized, integrated, and interpreted the interview transcripts until we found themes that described the self-reported experiences of the members of the LGBTQ+ community. Specifically, the researchers read the transcribed data several times and familiarized themselves with the data and made sure the data conformed to what was audio-taped. The researchers then proceeded to code the data, creating initial codes that captured significant portions of information within the content. The researchers diligently looked for patterns, connections, and recurring ideas. These recurring patterns and ideas provided the foundation for identifying the themes. These themes were carefully reviewed and scrutinized to see whether the themes accurately and comprehensively reflect the nuances and depth of the data. Each validated theme was then precisely defined and given a descriptive name. These well-defined themes were eventually used to write the results section. Throughout the analytical journey, the researchers remained vigilant about their own biases, assumptions, and positions. Acknowledging and reflecting on these factors ensured transparency and credibility of the analysis.

### Ethics approval and consent to participate

The researcher obtained ethical clearance from Institutional Review Board (IRB) of Noguchi Memorial Institute for Medical Research, University of Ghana, Legon (Protocol Approval Number: NMIMR-IRB CPN 059/23–24). Written informed consent was also obtained from each participant before recruitment into the study. Each participant was informed of his/her right to withdraw from the study at any time without suffering any negative consequences. Names of the participants were not revealed in the study report and all information gathered from the study participants were treated with outmost confidentiality as special codes were used to represent the responses of each participant.

## Results

### Socio-demographic characteristics

A total of fifteen self-confessed LGBTQ+ persons were interviewed in this study. Most of the Respondents were Christians with all of them having at least basic education. Regarding the sexual orientation and gender identity (SOGI) of the participants, seven (7) were bisexuals, five (5) were gays, one (1) was transgender and two (2) were lesbians. There were no queer or questioning participants among the fifteen (15) participants who participated in the study. Table 1 provides the details of socio-demographic characteristics of the participants.

**Table 1:** Socio-demographic characteristics of the participants.

Participant (Pseudonyms)	Gender Identity/Sexual Orientation	Marital Status	Religion	Occupation	Level of Education
CM1	Transgender	Not Married	Christian	Student	Tertiary (University Student)
CM2	Gay	Not Married	Christian	Program Officer (NGO)	Tertiary
CM3	Bisexual	Not Married	Christian	Fashion Designer	SHS
CM4	Bisexual	Not Married	Christian	Research Assistant	Tertiary
CM5	Lesbian	Not Married	Christian	Sales person in a bakery	JHS
CM6	Bisexual	Not Married	Muslim	Driver	JHS
CM7	lesbian	Not Married	Christian	Teacher (NSS)	Tertiary
CM8	Gay	Not Married	Christian	Student	Tertiary (University student)
CM9	Bisexual	Not Married	Christian	Student	Tertiary
CM10	Bisexual	Not Married	Muslim	SHS drop Out (Not working)	SHS (drop out)
CM11	Gay	Not Married	Muslim	University dropout	University drop out
CM12	Gay	Not Married	Christian	Student	Tertiary
CM13	Gay	Not Married	Christian	SHS dropout (learning graphic designing)	SHS
CM14	Bisexual	Not married	Christian	Teaching	Tertiary
CM15	Bisexual	Not married	Christian	IT	Tertiary

### Self-reported life experiences of members of the LGBTQ+ community

The participants recounted their experiences within their individual families, within the LGBTQ community, and their experiences with the public. The data analysis yielded one main theme and sub-themes. Table 2 presents the details of the major theme and sub-themes generated from the data.

**Table 2:** Synthesis of theme and sub-themes.

S/N	THEMES	SUBTHEMES
1	Life in the family	a. Challenges with family acceptance b. Threats of rejection c. Stigma
2	Life within the LGBTQ+ community	a. Vital source of support b. Identity c. Solidarity
3	Life in public	a. Stigma in society b. Discrimination c. Disapproval

### Experiences within their individual family

Under this main theme of life in the family, the participants recounted the diverse family dynamics they encounter within their individual families including challenges with family acceptance, threats of rejection and stigma. Thus, the following sub-themes emerged: challenges with family acceptance, threats of rejection and stigma

### Challenges with family acceptance

The participants claimed that they had challenges getting their family members to accept their sexual orientation and that their family members disapproved of them identifying as LGBTQ+ persons. For instance, some participants explained that their parents will be disappointed in them if they found out that they were into LGBTQ + , and that some of them were disowned by their families.

Below are some quotes from the participants

*“if my parents should find out what like I am actually into, like I don’t know what they will do, they could even disown me, they will be disappointed, for me my elder brother knows, he is a Nurse so he knows, he is okay with that, he is only cautioning me to be cautious of the people I engage with because of the prevalence of STIs and other diseases.” (CM 2 gay).*

*“My family members now know that I am in this practice, and they don’t want to be associated with me, my wife left me with the children when she got to know of it” (CM 3 bisexual).*

*“I cannot visit my family home, they hate me because of finding out what I do… hmmm, no family wants to be associated with me” (CM 5 lesbian)*

Another participant who identifies as a gay also explained how he is the only boy among his father ‘s children and some of his siblings do not approve of his LGBTQ+ community membership because they expect him to marry and produce children to continue their family lineage. He said:

*“Because currently my sister who doesn’t want me to be so, because we are only four siblings three girls and myself, so they don’t understand the fact that their only brother is going to be so. That one won’t be entertained, because they believe they will get married and their sir names will be changed and then I must make family to bear my Daddy’s name, so they won’t understand” (CM 13 gay).*

Another participant narrated how the mother loves him, and he has to keep telling lies to maintain that relationship anytime the mother asked him who his girlfriend is so as to be accepted by his mother and that his other family members disapproved of same. This is what he said:

*“…but because of my Mum I didn’t want to disappoint my Mum and all of that, and my Mum too she always ask me so who is your girlfriend now, who is she, so who is that and me when I talk to her she listens and I will be like she knows the situation in which we are now and me I want to make money before I think of marriage and all of that, so sometimes if my aunties say eiiiiii so you won’t come and show us our in-law and all of that, she will just say you guys should leave my son alone, he is making money if he makes money you people will see her” (CM 13 gay)*

*Similarly, a participant stated*

*“...because my mother was supporting what I was doing, and sees me as her daughter she is even the one who pierced my ears for me, but no one cares about me in my family” (CM 11 lesbian)*

### Threats of rejection

The participants also claimed that most of them were under threat of rejection from their family members and this made them stay away from their family members or not to disclose their sexual identities or preferences. Below are some quotes from the participants.

*“...I fear my family will reject me if they know I am in this practice, so I hide and do my things” (CM 2 gay)*

*“my father threatened me not to come home again if I will not change my sexual desires” (CM 11 lesbian)*

*“it is not easy to live this life in Ghana, your own family don’t want to see you and accept you the way you are. They even threaten to kill you if you let people know you are their family member, this is not fair to us” (CM 5 lesbian)*

*“I have lost my brothers and sisters, and my mother keeps sending me messages that if I don’t change then I should not call her mother” (CM 13 gay)*

### Stigma from family members

The participants said they were stigmatized and treated as unwanted by their family members. These are some of the quotes related to the stigma they faced in their families:

*“my family does not want me again, now the family claims that I have insulted the family name and have warned me from using the family name” (CM 5 lesbian).*

*“We are stigmatized because of this practice, and no one wants to be associated with me” (CM 15 bisexual).*

*“Our family does not want us again due to this our practice, I have no one to talk to as a family” (CM 8)*

### Experiences within the lgbtq+ community

The analysis revealed that the LGBTQ+ community itself served as a vital source of support, identity, and solidarity. The key sub-themes that emerged were vital sources of support, identity, and solidarity

### Vital sources of support

The data reveled that the LGBTQ+ community served as a vital source of support for the participants in their challenging times in the society. The participants stated that the LGBTQ+ community provides support to them when they are in need and even linked them up to friendly health facilities where they felt safe and received comprehensive care for their health challenges.

Below are some quotes from the participants

*“I could not have survived without the LGBTQ+ community, they provide us with guidance and support. At a point, the pressure was too high, and I had to marry a heterosexual to just keep to the societal pressure but the LGBTQ+ community was my game changer, supporting me throughout the process” (CM 15 bisexual).*

*“sometimes you want someone to talk to after all the rejections and my colleagues here are a good source of support” (CM 13 gay)*

*“it is only among ourselves that you are not judged but everyone in this country thinks we are not normal” (CM 5 lesbian)*

Some even claimed that the LGBTQ+ community provided them with financial support as indicated in the below quotes:

*“I lost my job because of my orientation, and it was my partner who supported me financially till I got another job” (CM 5 lesbian)*

*“my family refused to help me when I confessed my feelings to my dad and I depend on benevolent persons in this community for help ” (CM 11 gay)*

*“I have become financially independent due to the assistance I get from my partner, I use to suffer to provide my needs but now I am financially stable” (CM 12*)

A participant shared how supportive organizations help community members access health service with ease.

He stated

*“Okay so we have community-based organizations that are into easier health assessment for community members, so they can give you numbers of facilities where they have trained Nurses, and you can just go talk to them and then they will help you.” (CM 12 gay)*

Another participant also revealed how community-based NGOs even organize workshops for community members even though patronage is always low because of fear of stigma.

He said

*“Yeah it is something that is being done, but majority of the members don’t attend the workshops and things that we do, all because they know that if I come and this person sees me, maybe they will go and say that I am also part of the community, I don’t know if you get what I am saying?” (CM 2 gay)*

A participant also noted how they even have community members who are peer educators specifically for community members but some of the community members are not aware; He noted:

*“For the health service some of them don’t know that we have peer educators for community members, community members don’t know so they go to the hospital on their own, that one they charge you according to your problem.” (CM 3 bisexual)*

### Identity

The participants stated that the LGBTQ+ community was a useful community that recognized and accepted their sexual identities unlike the wider Ghanaian community that did not accept their sexual identities. Below are some quotes from the parties:

*“I have often been mocked in society due to the way I talk but I was accepted and encouraged among the LGBTQ*± *community to keep my uniqueness” (CM 13 gay)*

*“I know my identity in this life due to the LGBTQ*± *community” (CM 7 lesbian)*

*“You become accepted in this community and your sexual orientation and feeling is not seen as a problem but an identity as you have other people who are like you” (CM 11 gay)*

### Solidarity

The participants shared experiences that showed how there is mutual support and solidarity within the community, especially in time of need. They recounted instances when community members stood in to defend them and even be with them at crucial times. They appreciated the solidarity enjoyed withing the LGBTQ+ community.

This is supported by the below quotes

*“The only family I have now is the LGBTQ+ community because they are ready to stand-in and protect me against abuse and stigma” (CM 11 gay)*

*“The members stand for each other and go the full length to protect us and so I am happy with the community life among us” (CM 13)*

*“if not for this community, some of us would have been exposed because we would not even know there are special clinics exist for us and as for the general clinics, they will quickly expose you” (CM bisexual)*

*“The community provides support for members of the community and this helps us to cope with the neglect of society.” (CM 3 bisexual)*

### Experiences in public life

Participants expressed how navigating public life as an LGBTQ+ individual presents its own set of challenges. The analysis examined the diverse experiences of LGBTQ people in the broader society as they encountered varying degrees of stigma in society, discrimination, and disapproval

### Stigma in society

The participants claimed they were stigmatized due to their sexual orientation by the general society in Ghana. This stigma affected their social interactions and mental health. Below are some quotes from the participants.

*“The public stigmatize against us as if we are less humans, they view us as abnormal, and this makes us depressed and withdrawn from society” (CM 11 gay)*

*“Everyone seem to be against us and no one wants us to be associated with them so we stay in our quite places and this can be sickening” (CM 5 lesbian)*

A participant who identified as a transgender expressed her disquiet about how police arrested her and her colleagues and collected their money for identifying as transgender. She recounted:

*“I think somewhere last two years, my friends and I were arrested by some police officers, for identifying as trans, so they arrested us and took money from us which was defamation and crime, why are they taking money from us for being who we are and they have stolen our money from us, so that is it.” (CM 1 transgender)*

Another participant who identified as a gay also expressed his fear of stigma and as a result of that he is scared to go to church. He is afraid pastors may prophesize about his membership in the community and this affected their social networks. He explained:

*“So at a point because of what I am doing, I am scared to go to church because maybe a*

*Prophet will see something…a Prophet will be like young boy come, why are you doing this and that and the person will reveal what I am doing to the public and all of that, so I was scared to go to church, so at a point my Mum didn’t understand the reason let’s go to church I will say she should take the lead I will come, then I won’t come, she should take the lead I will come, then I won’t come and I prayed over it severally but then it is what it is, and then I gathered some courage I started going to church and anytime I am being called by a Prophet or something no Prophet has ever made mention about it before, they talk about something else and all of that so I started becoming comfortable and all of that.” (CM 13 gay)*

While one also expressed his disquiet about community members who are stigmatized and bullied in schools, homes and even hospitals. Some are even denied food and kicked out of their homes. He narrated:

*“The stigma in the society and even sometimes too at the hospital the bullying too, for me I have not experienced such before I think is because I am bi-sexual that is why, yeah so I have not experienced bullying before but some people go through bullying at schools, homes even some of them their own family members kick them out from their house, they don’t give them food they don’t give them place to sleep and they will be on the street struggling for their lives, that thing is not good, is not very good.” (CM 3 bisexual)*

### Discrimination

The participants claimed they were discriminated against by the public in Ghana. Some of them claimed they lost their jobs because of their sexual orientation and perceptions.

Below are some quotes from the participants

*“sometimes, people look at us and tag us and they discriminate against us for not being like them” (CM 1)*

*“I left my job due to how the people often looked at me as if I am an outcast of a sought” (CM 5 lesbian)*

Another participant who identifies as a bisexual also shared how he encountered homophobic threats from someone online through his dating App. He said:

*“….and he said that he is a homophobic and he is against this one and he want to catch this people around Adenta so that is why he introduced himself on the App and he is tracking them and they will beat you, that is what he said and I got shocked and even afraid for my life at that time, that was early last month he sent a picture and I also sent mine and he said that he knew me and he will let people beat me, what I am doing is it good? why should I myself like that, he is a homophobic and he swear he is tracking some guys around and then beat people who are into this so I was afraid and I am having a friend who told me to be careful, he sent me a video where they caught a community member and they were beating him naked, so for now I am really afraid even when I am doing, I am doing it low like even now I am not on any App, I am not on any dating App I am meeting the people that I know that is what I am doing now, I am not on any App.” (CM 9 bisexual)*

### Disapproval

The participants claimed the public disapproved of their sexual orientation and that they experience several instances of disapproval by just even their appearance and outlook in public. These were supported by the below quotes:

*“We know the society does not endorse what we do so we do our things in private so we don’t attract disapproval” (CM 5 Lesbian)*

*“The public are hostile towards us, even mere suspicion that you belong to this our group means that people disapprove of everything you do even if you do it well” (CM 12 gay)*

*“The culture of the country does not accept variety and so they disapprove of what we do, and it hurts so much” (CM 6 bisexual)*

A participant also explained how he is very discrete and mindful of his environment because the Ghanaian society is intolerant and hostile towards the community members. He explained how a community member was beaten in public transport (trotro) just for how he talks. He explained:

*“I don’t even know where to start from, you see, African society specifically Ghanaian society we are very hostile towards this thing, we are intolerant, in fact we don’t even want to have a room for discussion for it because we are being brain washed to understand that it is a foreign import, it is not a foreign import, it is not something that is being forced on us, it has been with us since time immemorial and even before the colonizers who are now being accused of introducing this came, but for me, I am being discrete as possible and I am taking charge of my own security, I am being mindful of my environment, I know how to behave wherever I find myself, because there is an instance where someone has been beaten in trotro just because of how the person talks.” (CM 2 gay)*

One also noted

*I think I get some difficulties at work, especially with my customers, yes with my customers some are not comfortable with my appearance and then with my look. They always complain about my look, they always complain about how I dress to work since they see me as a guy. (CM 5 lesbian)*

A participant also narrated how he always gets scared anytime parliament is deliberating on the LGBTQ+ laws. He stated:

*“I am afraid, I am very, very afraid whenever I see or hear parliament having issues over this and they are putting pressure and I think law is law so if they pass it and it becomes a law, I don’t think the community can do something to protect us” (CM 9 bisexual).*

The participants also claimed that the Ghanaian public were so hostile as compared to other jurisdictions and that some of them had plans of seeking asylum in other countries. Below are some quotes to support the participant’s case:

*“I have been insulted by people in this country due to my sexual affiliation, so I strongly want to relocate to another country”* (CM 8 gay).

*“Sometimes the people don’t care the way they talk to me in Ghana and they even use our gender preference to insult us, I wish I can seek asylum in the United States”* (CM 5 lesbian).

*“I want to leave to Europe, there they accept us better than in Ghana here*” (CM 7lesbian)

*“…..the public in Ghana hate us and will even kill us if they get to know what we do, recently they attacked some of our people and so I am hoping to leave at the least opportunity to a safer place like Spain or US”* (CM 11 gay)

## Discussion

The study uncovered diverse family dynamics, including varying degrees of acceptance and rejection. Many faced challenges with family expectations and pressures, particularly relating to marriage and lineage. Some participants received conditional support from family members, often contingent upon secrecy and maintaining traditional family roles. This revelation of the study is in alignment with existing literature [2,3] which supports these findings, indicating that LGBTQ individuals globally face similar challenges within their families. These studies [2,3] have also shown that family acceptance can significantly impact mental health and wellbeing, while rejection or conditional acceptance can lead to adverse outcomes, including depression, anxiety, and increased risk of suicidal ideation and suicide. The health implications of family rejection are significant [2,7,10,11]. Chronic stress, often a result of family rejection, can trigger mental health disorders such as anxiety and depression [7,11]. Additionally, the stress associated with navigating family rejection or conditional acceptance can lead to adverse health outcomes beyond mental health, including cardiovascular disease and substance abuse [6,10,11]. The link between family dynamics and health outcomes underscores the urgent need for interventions aimed at fostering family acceptance of LGBTQ individuals [6]. Family-based interventions and public health campaigns focused on education about LGBTQ+ identities could be critical in mitigating these negative health outcomes by promoting acceptance and understanding within families [1,2].

In contrast to the challenges faced within family settings, the LGBTQ+ community itself serves as a vital source of support, solidarity, and resilience. The study found that community-based organizations including NGOs interested in LGBTQ+ welfare and peer educators play an instrumental role in providing LGBTQ individuals with access to healthcare, social services, and safe spaces for self-expression. However, the fear of stigma and exposure was cited as a deterrent to fully engaging with these community resources, despite their critical role in providing support. This finding aligns with previous studies, which emphasized the importance of community networks in promoting the well-being of LGBTQ individuals. Studies by [8,13,14] highlight how LGBTQ support groups and community organizations facilitate access to essential services, such as healthcare, legal assistance, and mental health resources. These community-based networks provide a refuge from the discrimination and stigma often encountered in mainstream society, helping LGBTQ individuals to navigate health systems and access culturally competent care without fear of exposure or mistreatment [7,14–18].

Participants in this study consistently emphasized the role of peer educators and community organizations in connecting them to healthcare service providers that are knowledgeable and affirming of their needs. This connection is vital in contexts where mainstream healthcare services are often inaccessible or unwelcoming to LGBTQ+ individuals due to stigma, discrimination, or a lack of provider competence in LGBTQ+ health issues. By bridging the gap between the LGBTQ community and healthcare providers, these organizations help to mitigate the negative health impacts of societal discrimination, such as delayed care, unmet health needs, and increased vulnerability to mental and physical health challenges [1,6,19–21]. The ability of community-based organizations to reduce health disparities cannot be overstated especially in environments where LGBTQ individuals may avoid mainstream healthcare due to fear of mistreatment, these organizations provide a lifeline, offering trusted sources of information and advocacy. The role of community support networks in facilitating access to healthcare is particularly important in ensuring that LGBTQ+ individuals receive timely and appropriate care, thus improving health outcomes and reducing the overall burden of health disparities in the LGBTQ+ community [6].

The study revealed significant challenges faced by LGBTQ individuals in public life, including widespread discrimination, violence, and stigmatization. Participants reported experiences of police harassment, threats from homophobic individuals, and fear of exposure in both online and physical spaces, particularly religious and public settings. These findings are consistent with the broader body of literature on the experiences of LGBTQ individuals, who often face societal intolerance and systemic discrimination [6,7]. The pervasive nature of discrimination against LGBTQ individuals is a significant contributor to health disparities within this community. Experiences of violence and harassment can lead to severe mental health challenges, including post-traumatic stress disorder (PTSD), depression, and anxiety [2]. Studies have shown that LGBTQ individuals are disproportionately affected by mental health issues, often exacerbated by the societal stigma and lack of legal protections that compound their vulnerability [15–17,21].

The lack of legal protections for LGBTQ individuals+ in many societies further exacerbates this issue, as participants in this study reported fear of reporting incidents of harassment or violence to authorities due to potential backlash or further victimization. This contributes to a cycle of vulnerability, where LGBTQ individuals are left with few avenues for recourse or protection, further deepening their health disparities [7,15–17]. The findings from this study underscore the need for comprehensive legal reforms and public health interventions that address the systemic discrimination faced by LGBTQ individuals. Legal protections, anti-discrimination laws, and societal education campaigns are critical to creating an environment where LGBTQ individuals can live without fear of violence or harassment [6,17–21]. Additionally, healthcare systems must be equipped to address the unique health challenges resulting from societal discrimination, offering mental health support and trauma-informed care that recognizes the impact of public life on LGBTQ individuals’ well-being [3,16].

The findings from this study reveal the complex and multifaceted experiences of LGBTQ individuals, shaped by family dynamics, community support, and public life challenges. The mental and physical health of LGBTQ individuals is significantly influenced by their interactions with family, community, and society at large [3,17]. While family rejection or conditional acceptance poses significant mental health risks, community-based support networks provide a critical buffer against these challenges by facilitating access to healthcare and safe spaces for self-expression. However, societal discrimination and public life challenges continue to pose substantial barriers to the well-being of LGBTQ individuals, contributing to widespread health disparities [15–17]. These findings highlight the importance of a holistic approach to improving the health and wellbeing of LGBTQ individuals, one that addresses the family, community, and societal factors that shape their experiences so as to ensure more inclusivity [15–21].

### Limitations of the study

The results of this study can only speak to the limited population studied who were residents of Accra. Therefore, the results cannot be generalized for the entire LGBTQ+ population in Ghana particularly non-residents of Accra. However, the findings provide in-depth data on the subject matter and provide a basis for future representative studies.

The use on non-probability sampling techniques could have biased the findings of this study. However, such sampling techniques are appropriate for the specific design used for this study, which was qualitative in nature and not aimed at generalization hence no need for probability sampling technique.

This study was limited to the general members of the LGBTQ+ community members. Future studies could explore and compare the experiences of the various categories of the LGBTQ+ so as to make specific recommendations for each specific group.

## Conclusion

The participants claimed that the general society (public) in Ghana stigmatizes against members of the LGBTQ+ community with most families rejecting members who claim to be LGBTQ+ persons. The participants however claimed that the LGBTQ+ community served as a major source of support for them and helped them cope with the rejection and stigma associated with their sexual orientations. They further claimed that the stigma and rejection contributed to poor mental health of most LGBTQ+ persons with most of them seeking asylum in more inclusive and welcoming jurisdictions.

We recommend that the Government of Ghana through the Ministry of Gender, Children and Social Protection and the Ghana Health Service increases public education to improve cultural competence and inclusivity of this vulnerable population in society to reduce stigma and discrimination.

## Supporting information

S1 File Interview guide. (DOCX)

## Abbreviations

LGBTQ: Lesbian, Gay, Bisexual, Transgender, Queer/Questioning
MSM: Men who have sex with men
SOGI: Sexual Orientation and Gender Identity
SSA: Sub Saharan Africa
