# Supplementary material for: Self-reported life experiences of members of the LGBTQ+ community in Accra, Ghana
Source: PLoS One. 2025 May 29;20(5):e0325148. doi: 10.1371/journal.pone.0325148 (PMC12121748; doi:10.1371/journal.pone.0325148)
Supplement: S1 File — (DOCX) [file pone.0325148.s001.docx]

**INTERVIEW GUIDE**

**DATA COLLECTION INSTRUMENTS**

This current study titled “Self-reported life experiences of members of the LGBTQ+ community in Accra Ghana” is part of the study titled “Exploration of LGBTQ community members’ experiences with utilization of healthcare services in the Accra Metropolis” in which ethical approval has been obtained.

**SECTION A**

**INTRODUCTION AND DEMOGRAPHIC DATA**

How old are you now?

Which gender identity do you possess or consider yourself to belon?

What is your specific sexual orientation?

Kindly let us talk about your educational level?

What specific job do you do (Occupation)?

**SECTION B**

**MAIN INTERVIEW SESSION**

**LIFE EXPERIENCES OF MEMBERS OF THE LGBTQ+ COMMUNITY IN ACCRA, GHANA.**

1.How is life in a family as an LGBTQ+ community member in Ghana?

Probes:

- Are there any specific situations in your family that you have experienced due to your sexual orientation? Please Explain

• Have there been any changes in the way you are seen by your family due to your sexual orientation? Please Explain

2. How is life in the LGBTQ+ community member in Ghana?

Probes:

- Tell me about how the LGBTQ+ community help members? Please Explain

• How does the LGBTQ+ community assist LGBTQ+ community members? Please Explain

3. Please share with me your personal experiences in public with being a member of the LGBTQ+ community in Ghana?

Probes:

• How does society see you due to your sexual oreientation?

• Please provide an example of a some recent public issues that you experienced because you are a member of the LGBTQ+ ?

**Thank you for participation**
