# Supplementary material for: Self-reported life experiences of members of the LGBTQ+ community in Accra, Ghana
Source: PLoS One. 2025 May 29;20(5):e0325148. doi: 10.1371/journal.pone.0325148 (PMC12121748; doi:10.1371/journal.pone.0325148)
Supplement: Attachment — Submitted filename: Response to Reviewers.pdf [file pone.0325148.s004.pdf]

University of Ghana
College of Health Sciences
School of Nursing and Midwifery
Department of Adult Health

20th April, 2025

The Editor
PLOS ONE

Dear Sir/Madam,

RESPONSE TO REVIEWERS

We are grateful for the review of our manuscript titled "**Self-reported life experiences of members of the LGBTQ+ community in Accra, Ghana**" to make it better for possible publication.

Comments from the editor

Journal Requirements:
When submitting your revision, we need you to address these additional requirements.

1.Please ensure that your manuscript meets PLOS ONE's style requirements, including those for file naming. The PLOS ONE style templates can be found at https://journals.plos.org/plosone/s/file?id=wjVg/PLOSOne_formatting_sample_main_body.pdf and https://journals.plos.org/plosone/s/file?id=ba62/PLOSOne_formatting_sample_title_authors_affiliations.pdf

2. We note that your Data Availability Statement is currently as follows: All relevant data are within the manuscript and in Supporting Information files.
Please confirm at this time whether or not your submission contains all raw data required to replicate the results of your study. Authors must share the "minimal data set" for their submission. PLOS defines the minimal data set to consist of the data required to replicate all study findings reported in the article, as well as related metadata and methods (https://journals.plos.org/plosone/s/data-availability#loc-minimal-data-set-definition).
For example, authors should submit the following data:
- The values behind the means, standard deviations and other measures reported;
- The values used to build graphs;
- The points extracted from images for analysis.
Authors do not need to submit their entire data set if only a portion of the data was used in the reported study.
If your submission does not contain these data, please either upload them as Supporting Information files or deposit them to a stable, public repository and provide us with the relevant URLs, DOIs, or accession numbers. For a list of recommended repositories, please see https://journals.plos.org/plosone/s/recommended-repositories.
If there are ethical or legal restrictions on sharing a de-identified data set, please explain them in detail (e.g., data contain potentially sensitive information, data are owned by a third-party organization, etc.) and who has imposed them (e.g., an ethics committee). Please also provide contact information for a data access committee, ethics committee, or other institutional body to which data requests may be sent. If data are owned by a third party, please indicate how others may request data access.

3. Please include your full ethics statement in the ‘Methods’ section of your manuscript file. In your statement, please include the full name of the IRB or ethics committee who approved or waived your study, as well as whether or not you obtained informed written or verbal consent. If consent was waived for your study, please include this information in your statement as well.

4. Please review your reference list to ensure that it is complete and correct. If you have cited papers that have been retracted, please include the rationale for doing so in the manuscript text, or remove these references and replace them with relevant current references. Any changes to the reference list should be mentioned in the rebuttal letter that accompanies your revised manuscript. If you need to cite a retracted article, indicate the article’s retracted status in the References list and also include a citation and full reference for the retraction notice.

[Note: HTML markup is below. Please do not edit.]

Response to editor

1. We have complied with the requirements for file naming as suggested by the editor.
2. We have complied with the suggestions on data availaibility by providing the recommended data availability statement in the manuscript. This is on page 21 of the revised manuscript.
3. We have included the full ethics statement in the methods section of the revised manuscript . This can be found on page 9 of the revised manuscript.
4. We have revised the reference list to make it complete as suggested by the reviewer. This can be found on pages 21 to 23 of the revised manuscript

Editor’s comments

hope this message finds you well. Thank you for submitting your manuscript titled "**Self-reported life experiences of members of the LGBTQ+ community in Accra, Ghana**" to our journal. After careful review, we have determined that your manuscript requires minor revisions before we can proceed with further decisions. Below, I have outlined the revisions/improvements needed:

1. Abstract

- Revise the **Results** section:
    - Replace “other geographical settings” with “within their families” to align with the identified themes.
    - Alternatively, clarify what "other geographical settings" refers to.
- Revise the **Conclusion** section:
    - Clearly indicate the target audience for the recommendations (e.g., government, policymakers, global community).

2. Introduction

- Clearly **state the research gap** and the specific problem being addressed.

- **Introduce "life experiences" earlier** in the introduction to establish its central role in the study.
- **Engage with relevant literature** on life experiences of LGBTQ+ individuals to justify the study's significance.
- **Connect policies and laws** (macro level) to the lived experiences (micro level) of LGBTQ+ individuals.
- Ensure **smooth transitions** between ideas, particularly before the sentence: "There is little information in the literature on the life experiences of LGBTQ+ persons in the Ghanaian society."

3. Methods

3.1 Study Setting

- Briefly discuss **gender and sexual orientation context** in Ghana to provide a deeper understanding of the setting.

3.2 Population, Sampling, and Sample Size

- Ensure all details related to participant selection and sample size are contained in this section.
- Clarify **how participants were recruited**, especially if they were recruited in clinics.
    - Address **ethical considerations** related to participant recruitment, particularly concerning safety and security.
    - Specify whether participants were **informed beforehand** and how access to them was gained.
- Move the sentence about **data saturation** ("Data collection from participants was done until no new information emerged.") to the **Data Collection** section.

3.3 Data Collection and Tools

- **Revise the section title** from "Selection of Participants and Data Collection" to **"Data Collection and Tools."**
- Indicate **where the interviews were conducted** and the rationale for selecting those locations to ensure a natural interview process.

3.4 Data Analysis

- **Add a section on Reflexivity** to discuss:
    - Researcher positionality, assumptions, and biases.
    - How power dynamics between researcher and participants were managed.
    - Measures taken to ensure credibility of analysis.

- Clarify the concept of **bracketing** and how it was applied, considering scholarly critiques on its feasibility.

3.5 Methodological Rigor

- Clearly define **transferability** in the study's context.
- Provide details on how bracketing was applied and its impact on the study.

4. Results

- Ensure **consistent allocation of participant quotes** (avoid attributing multiple quotes to a single participant).
- Add a **summary at the end of each theme** to synthesize key findings.
- **Revise Table 2** to ensure the overarching theme directly reflects LGBTQ+ experiences.

4.1 Life in the Family

- Ensure descriptions match the corresponding quotes (e.g., revise "Another participant narrated…" when multiple quotes follow).

5. Discussion

- The discussion is strong but ensure **integration of qualitative and quantitative findings** where applicable.

6. Conclusion

- Revise the conclusion to:
  - Clearly state **the significance and implications** of the study for policy and practice.
  - Provide **targeted recommendations** for specific audiences (e.g., government, policymakers, community organizations).

7. Limitations of the Study

- **Move this section before the conclusion** for logical flow.
- Ensure limitations are clearly stated, including:
  - The small sample size and its implications.
  - The potential influence of the study setting on findings.

8. Implications for Future Research

- Add a **specific section on future research** to:

    - Identify areas needing further study (e.g., larger-scale research, intersectionality, mental health impacts).

9. Author Contributions

- Include a **statement detailing each author's contribution** to align with journal requirements.

By addressing these comments, you can improve the comprehensiveness, relevance, and clarity of your research article.

Please address these revisions within 45 days, by or before May 7, 2025. If you have any questions or need further clarification on any of the points mentioned above, please do not hesitate to contact us.

Thank you for your attention to these revisions. We look forward to receiving the updated manuscript.

Best regards,

Mc Rollyn D. Vallespin
Academic Editor
PLOS ONE

Responses of Authors to Review

1. Abstract

- We have reviewed the results section of the abstract as indicated on page 2 of the revised manuscript:
    - We have edited the statement and removed "other geographical settings" as suggested by the reviewer
- Revise the **Conclusion** section:
    - We have stated the specific persons for which the recommendations are targeted as indicated on page 2 of the revised manuscript

2. Introduction

- We have identified the gap in the literature for which this study was conducted as suggested by the reviewer. This is found on pages 3 and 4 of the revised manuscript.

- We have introduced life experiences in the earlier part of the introduction of the manuscript as suggested by the reviewer. This is found on page 3 of the revised manuscript.
- We have justified the significance of the study with relevant literature as indicated on pages 3 and 4 of the revised manuscript.
- We have ensured a transition in ideas as suggested by the reviewer in the introduction. This is found on pages 3 and 4 of the revised manuscript.

3. Methods

3.1 Study Setting

- We have provided the specific gender dynamics and sexual orientation of the study setting as indicated on pages 4 and 5 of the revised manuscript.

3.2 Population, Sampling, and Sample Size

- We have provided information on participant selection and sample size in this section. This is indicated on page 5 of the revised manuscript.
- We have clarified how the participants were recruited as well as the specific way by which the researchers gained access to the study participants. This is found on pages 6 and 7 of the revised manuscript on data collection
- We have moved the information on data saturation to the data collection section on pages 6 and 7 of the revised manuscript.

3.3 Data Collection and Tools

- We have revised the section title from "Selection of Participants and Data Collection" to "Data Collection and Tools" as suggested by the review. This can be found on page 6 of the revised manuscript.
- We have indicated where the interviews were conducted in the revised manuscript on page 6 specifically at Data collection and tool

3.4 Data Analysis

  - We have included a section on reflexivity as suggested by the reviewer. This is found on page 8 of the revised manuscript. We have discussed our positionality, assumptions, and biases as well as the power dynamics between researcher and participants were managed.
  - We have further discussed measures used to ensure credibility of the findings in the revised manuscript as indicated on pages 7 and 8 of the revised manuscript.

- We have discussed bracketing of our experiences under methodological rigour discussed on page 7 of the revised manuscript.

3.5 Methodological Rigor

- We have discussed the issues of bracketing in the revised manuscript on page 7.

4. Results

- We have ensured consistent allocation of participant quotes as suggested by the reviewer throughout the results section.
- We have revised Table 2 pursuant to the suggestions of the review. This can be found on page 11 of the revised manuscript.

.

4.1 Life in the Family

- We have revised the quotes under this section to reflect the directions of the reviewer.

5. Discussion

- We have re-written the discussions as suggested by the reviewer. This can be found on pages 17 to 20 of the revised manuscript.

6. Conclusion

- We have revised the conclusion of the study and clearly stated the implication of the findings for policy makers. This can be found on page 20 of the revised manuscript.

7. Limitations of the Study

- We have moved the limitation to now come before the conclusion as suggested by the reviewer. This can be found on page 20 of the revised manuscript.
- We have discussed specific limitations related to sampling and the setting on the findings as suggested by the reviewer. This can be found on page 20 of the revised manuscript.

8. Implications for Future Research

  - We have discussed implications for future research in the limitations section of the revised manuscript on page 20.

9. Author Contributions

- We have included the contributions of the authors to the revised manuscript as suggested by the reviewer on page 21 of the revised manuscript

We hope our manuscript would be considered for publication.

Thank you

Yours sincerely,
Dr. Kennedy Dodam Konlan
(Corresponding author)
