# Supplementary material for: Self-reported life experiences of members of the LGBTQ+ community in Accra, Ghana
Source: PLoS One. 2025 May 29;20(5):e0325148. doi: 10.1371/journal.pone.0325148 (PMC12121748; doi:10.1371/journal.pone.0325148)
Supplement: Attachment — Submitted filename: MAR 23 2025-Konlan.docx [file pone.0325148.s002.docx]

Subject: Minor Revisions Required for Your Manuscript Submission

Dear Dr. KONLAN,

I hope this message finds you well. Thank you for submitting your manuscript titled "**Self-reported life experiences of members of the LGBTQ+ community in Accra, Ghana**" to our journal. After careful review, we have determined that your manuscript requires minor revisions before we can proceed with further decisions. Below, I have outlined the revisions/improvements needed:

**1. Abstract**

- Revise the **Results** section:
  - Replace “other geographical settings” with “within their families” to align with the identified themes.
  - Alternatively, clarify what "other geographical settings" refers to.
- Revise the **Conclusion** section:
  - Clearly indicate the target audience for the recommendations (e.g., government, policymakers, global community).

**2. Introduction**

- Clearly **state the research gap** and the specific problem being addressed.
- **Introduce "life experiences" earlier** in the introduction to establish its central role in the study.
- **Engage with relevant literature** on life experiences of LGBTQ+ individuals to justify the study’s significance.
- **Connect policies and laws** (macro level) to the lived experiences (micro level) of LGBTQ+ individuals.
- Ensure **smooth transitions** between ideas, particularly before the sentence: “There is little information in the literature on the life experiences of LGBTQ+ persons in the Ghanaian society.”

**3. Methods**

**3.1 Study Setting**

- Briefly discuss **gender and sexual orientation context** in Ghana to provide a deeper understanding of the setting.

**3.2 Population, Sampling, and Sample Size**

- Ensure all details related to participant selection and sample size are contained in this section.
- Clarify **how participants were recruited**, especially if they were recruited in clinics.
  - Address **ethical considerations** related to participant recruitment, particularly concerning safety and security.
  - Specify whether participants were **informed beforehand** and how access to them was gained.
- Move the sentence about **data saturation** ("Data collection from participants was done until no new information emerged.") to the **Data Collection** section.

**3.3 Data Collection and Tools**

- **Revise the section title** from "Selection of Participants and Data Collection" to **"Data Collection and Tools."**
- Indicate **where the interviews were conducted** and the rationale for selecting those locations to ensure a natural interview process.

**3.4 Data Analysis**

- **Add a section on Reflexivity** to discuss:
  - Researcher positionality, assumptions, and biases.
  - How power dynamics between researcher and participants were managed.
  - Measures taken to ensure credibility of analysis.
- Clarify the concept of **bracketing** and how it was applied, considering scholarly critiques on its feasibility.

**3.5 Methodological Rigor**

- Clearly define **transferability** in the study’s context.
- Provide details on how bracketing was applied and its impact on the study.

**4. Results**

- Ensure **consistent allocation of participant quotes** (avoid attributing multiple quotes to a single participant).
- Add a **summary at the end of each theme** to synthesize key findings.
- **Revise Table 2** to ensure the overarching theme directly reflects LGBTQ+ experiences.

**4.1 Life in the Family**

- Ensure descriptions match the corresponding quotes (e.g., revise "Another participant narrated…" when multiple quotes follow).

**5. Discussion**

- The discussion is strong but ensure **integration of qualitative and quantitative findings** where applicable.

**6. Conclusion**

- Revise the conclusion to:
  - Clearly state **the significance and implications** of the study for policy and practice.
  - Provide **targeted recommendations** for specific audiences (e.g., government, policymakers, community organizations).

**7. Limitations of the Study**

- **Move this section before the conclusion** for logical flow.
- Ensure limitations are clearly stated, including:
  - The small sample size and its implications.
  - The potential influence of the study setting on findings.

**8. Implications for Future Research**

- Add a **specific section on future research** to:
  - Identify areas needing further study (e.g., larger-scale research, intersectionality, mental health impacts).

**9. Author Contributions**

- Include a **statement detailing each author’s contribution** to align with journal requirements.

By addressing these comments, you can improve the comprehensiveness, relevance, and clarity of your research article.

Please address these revisions within 45 days, by or before May 7, 2025. If you have any questions or need further clarification on any of the points mentioned above, please do not hesitate to contact us.

Thank you for your attention to these revisions. We look forward to receiving the updated manuscript.

Best regards,

**Mc Rollyn D. Vallespin**
Academic Editor
PLOS ONE
