# Supplementary material for: Self-reported life experiences of members of the LGBTQ+ community in Accra, Ghana
Source: PLoS One. 2025 May 29;20(5):e0325148. doi: 10.1371/journal.pone.0325148 (PMC12121748; doi:10.1371/journal.pone.0325148)
Supplement: Peer review history

# Author comment: Author response to Decision Letter 0

**DOI:** 10.1371/journal.pone.0325148.r001

28 Nov 2024

# Peer review: Decision Letter 0

**DOI:** 10.1371/journal.pone.0325148.r002

**Authors:** Daryl Higgins

16 Apr 2025

PONE-D-24-54894Self-reported life experiences of members of the LGBTQ+ community in Accra, GhanaPLOS ONE

Dear Dr. Konlan,

Thank you for submitting your manuscript to PLOS ONE. After careful consideration, we feel that it has merit but does not fully meet PLOS ONE’s publication criteria as it currently stands. Therefore, we invite you to submit a revised version of the manuscript that addresses the points raised during the review process.

==============================

**The reviews were very positive, seeing lots of merit with the article, but provide useful suggestions for improvements that I would encourage you to address thoroughly before resubmitting.**

==============================

Please submit your revised manuscript by May 31 2025 11:59PM. If you will need more time than this to complete your revisions, please reply to this message or contact the journal office at plosone@plos.org . When you're ready to submit your revision, log on to https://www.editorialmanager.com/pone/ and select the 'Submissions Needing Revision' folder to locate your manuscript file.

Please include the following items when submitting your revised manuscript:

- A rebuttal letter that responds to each point raised by the academic editor and reviewer(s). You should upload this letter as a separate file labeled 'Response to Reviewers'.
- A marked-up copy of your manuscript that highlights changes made to the original version. You should upload this as a separate file labeled 'Revised Manuscript with Track Changes'.
- An unmarked version of your revised paper without tracked changes. You should upload this as a separate file labeled 'Manuscript'.

If you would like to make changes to your financial disclosure, please include your updated statement in your cover letter. Guidelines for resubmitting your figure files are available below the reviewer comments at the end of this letter.

If applicable, we recommend that you deposit your laboratory protocols in protocols.io to enhance the reproducibility of your results. Protocols.io assigns your protocol its own identifier (DOI) so that it can be cited independently in the future. For instructions see: https://journals.plos.org/plosone/s/submission-guidelines#loc-laboratory-protocols . Additionally, PLOS ONE offers an option for publishing peer-reviewed Lab Protocol articles, which describe protocols hosted on protocols.io. Read more information on sharing protocols at https://plos.org/protocols?utm_medium=editorial-email&utm_source=authorletters&utm_campaign=protocols .

We look forward to receiving your revised manuscript.

Kind regards,

Daryl Higgins, PhD

Academic Editor

PLOS ONE

Journal Requirements:

When submitting your revision, we need you to address these additional requirements.

1.Please ensure that your manuscript meets PLOS ONE's style requirements, including those for file naming. The PLOS ONE style templates can be found at

https://journals.plos.org/plosone/s/file?id=wjVg/PLOSOne_formatting_sample_main_body.pdf and

https://journals.plos.org/plosone/s/file?id=ba62/PLOSOne_formatting_sample_title_authors_affiliations.pdf

2. We note that your Data Availability Statement is currently as follows: All relevant data are within the manuscript and in Supporting Information files.

Please confirm at this time whether or not your submission contains all raw data required to replicate the results of your study. Authors must share the “minimal data set” for their submission. PLOS defines the minimal data set to consist of the data required to replicate all study findings reported in the article, as well as related metadata and methods (https://journals.plos.org/plosone/s/data-availability#loc-minimal-data-set-definition).

For example, authors should submit the following data:

- The values behind the means, standard deviations and other measures reported;

- The values used to build graphs;

- The points extracted from images for analysis.

Authors do not need to submit their entire data set if only a portion of the data was used in the reported study.

If your submission does not contain these data, please either upload them as Supporting Information files or deposit them to a stable, public repository and provide us with the relevant URLs, DOIs, or accession numbers. For a list of recommended repositories, please see https://journals.plos.org/plosone/s/recommended-repositories.

If there are ethical or legal restrictions on sharing a de-identified data set, please explain them in detail (e.g., data contain potentially sensitive information, data are owned by a third-party organization, etc.) and who has imposed them (e.g., an ethics committee). Please also provide contact information for a data access committee, ethics committee, or other institutional body to which data requests may be sent. If data are owned by a third party, please indicate how others may request data access.

3. Please include your full ethics statement in the ‘Methods’ section of your manuscript file. In your statement, please include the full name of the IRB or ethics committee who approved or waived your study, as well as whether or not you obtained informed written or verbal consent. If consent was waived for your study, please include this information in your statement as well.

4. Please review your reference list to ensure that it is complete and correct. If you have cited papers that have been retracted, please include the rationale for doing so in the manuscript text, or remove these references and replace them with relevant current references. Any changes to the reference list should be mentioned in the rebuttal letter that accompanies your revised manuscript. If you need to cite a retracted article, indicate the article’s retracted status in the References list and also include a citation and full reference for the retraction notice.

[Note: HTML markup is below. Please do not edit.]

Reviewers' comments:

Reviewer's Responses to Questions

**Comments to the Author**

1. Is the manuscript technically sound, and do the data support the conclusions?

The manuscript must describe a technically sound piece of scientific research with data that supports the conclusions. Experiments must have been conducted rigorously, with appropriate controls, replication, and sample sizes. The conclusions must be drawn appropriately based on the data presented. 

Reviewer #1: Yes

Reviewer #2: Yes

**********

2. Has the statistical analysis been performed appropriately and rigorously? 

Reviewer #1: N/A

Reviewer #2: Yes

**********

3. Have the authors made all data underlying the findings in their manuscript fully available?

The PLOS Data policy requires authors to make all data underlying the findings described in their manuscript fully available without restriction, with rare exception (please refer to the Data Availability Statement in the manuscript PDF file). The data should be provided as part of the manuscript or its supporting information, or deposited to a public repository. For example, in addition to summary statistics, the data points behind means, medians and variance measures should be available. If there are restrictions on publicly sharing data—e.g. participant privacy or use of data from a third party—those must be specified.

Reviewer #1: Yes

Reviewer #2: Yes

**********

4. Is the manuscript presented in an intelligible fashion and written in standard English?

PLOS ONE does not copyedit accepted manuscripts, so the language in submitted articles must be clear, correct, and unambiguous. Any typographical or grammatical errors should be corrected at revision, so please note any specific errors here.

Reviewer #1: Yes

Reviewer #2: Yes

**********

5. Review Comments to the Author

Please use the space provided to explain your answers to the questions above. You may also include additional comments for the author, including concerns about dual publication, research ethics, or publication ethics. (Please upload your review as an attachment if it exceeds 20,000 characters)

Reviewer #1: Many thanks to the authors for this study. The authors explored rampant issues the world is experiencing today. Social disapproval, inequality, stigma, and discrimination, among other adversities experienced by individuals or groups of individuals based on their social identities, are critical concerns in today’s world, as it has been. The experiences of social disapproval and stigma endured by the LGBTQ + community because of their gender identities and sexual orientation in Ghana documented in this study reflect the global inequalities, discrimination and oppression due to individual identities, which has a profound negative impact on the well-being of people worldwide. Besides sexual orientation and gender identities, other identities include race, ethnicity, poverty, and geographical location identities. Individuals with minor identities experience various hardships, fuelling health disparities and poor quality of life.

Also, this study underscores the importance of qualitative research in understanding complex experiences of the human race to inform policymakers, civil societies and community members. By exploring everyday experiences, the study shed light on how cultural, social structure and politics intersect to influence daily experiences of individuals/groups of people at different levels.

The authors have described the basic requirements for conducting qualitative research in the manuscript. However, there are areas the authors need to improve, and I have presented minor comments in the following sections and in the text:

Minor comments

• Abstract

The abstract provides a useful overview and the background of the study and covers the content of the study. However, there are areas in the results and conclusions sections that need to be revised.

In the result section where the authors wrote “...as well as other geographical settings”, the word “other” as it has an indefinite meaning, creates confusion, wondering what those other settings are. It would be better to focus on the experiences in the three areas you have reported–in family, public and LGBTQ+ community in this section.

I would suggest replacing it with “within their families” which is the third sub-theme identified in the study. Otherwise, they will need to elaborate more on those “other geographical settings” they referred to.

• Conclusion

The authors have provided recommendations, but it would be useful to indicate to whom they need to be directed, for example, the government, policymakers, or the global community this can help the use of the study findings.

• Introduction

The authors have given a good background on the situation of the acceptance of the LGBTQ+ community at global, regional and country levels. However, the authors did not clearly describe the current gaps and the problem they want to solve. I would recommend highlighting the gaps, the problem this study is trying to solve and the significance of this study to the LGBTQ+ community in Ghana, and at the global scale.

The term life experience is a central topic of this study, but it emerges at the end of the introduction, while it would have come earlier. The life experience should be placed in the very first paragraph for the reader to know what this study is about and give discuss it. The authors should engage with the literature on life experience in the introduction section and discuss why you want to explore it in relation to LGBTQ+ community, as this will help the authors to discuss their contribution and to ensure the integrity of the study.

The authors discussed a lot about laws and policies concerning LGBTQ+ in Ghana and elsewhere, it would be good to connect them [laws and policies] with the everyday life of LGBTQ+ community discussing how policies [at macro level] shape individual experience [micro level] justifying the significance of exploring the life experience of LBGTQ+ community to advocate for their inclusion in society.

Also, the sentence “There is little information in the literature on the life experiences of LGBTQ+ persons in the Ghanaian society.” emerges abruptly toward the end of the section of the introduction, it does not link with the previous sentence. It would be better to have a smooth transition.

• Methods

The authors have prettily described the methods and tools used in this study. However, there are a lot of repetitions and misplacement of contents. I would recommend revising the subtitles and placing the contents appropriately. My detailed comments are presented in the following section and the text:

Study setting

The description of the characteristics of a study setting is important in qualitative research, as it gives insights into the contextual information, which is essential in understanding the findings and can inform the transferability of the study findings. The authors have described and justified the geographical setting, which is very important. However, it would be useful to briefly talk about the background of gender and sexual orientation in the study setting to help readers understand the context and implication this can have on the findings.

Population, sampling and sample size.

All the information related to the selection of participants, and sample size should be covered in this section, if the authors want to keep this sub-title.

The authors said that they selected the first participant at a clinic while receiving treatment. I found an ethical breach in this sentence. Given that the security of the LGBTQ+ community members was at threat as described in the manuscript, it is important to discuss briefly how you got access to the participants and the ethical challenges faced during the selection of participants.

The authors need to explain whether the participant was informed beforehand, and how they got access to the participant at a clinic. Was the clinic a study site? If the authors selected participants from clinics, homes or other places, it would be good to describe those sub-settings and explain how they got access to the study participants.

Under this section, the authors wrote “Data collection from participants was done until no new information emerged [12]. The data saturation in this research was achieved after the 15th participant’s interview.” , this information would fit better under the data collection section.

Selection of participants and data collection

This section should cover the information related to data collection, not a mixture of selection of participants as this has been discussed under “Population, sampling and sample size section”. I would suggest revising this sub-title “Selection of participants and Data Collection as “Data collection and tools”, because the authors have already discussed the selection process in the previous section.

The authors mentioned the duration of the interview, which is important. Equally, knowing where the places interviews happened is important. I would recommend the authors indicate places where the interviews occurred and how the decisions on selecting interview locations were made to ensure that interviews were run naturally, which is critical in qualitative research.

Data analysis

The relationship between researcher and researched/study participants plays a significant role in qualitative research as it influences research in many ways, including data collection, analysis and interpretation process. Power dynamics and hierarchy that exist between researchers and participants influence the type of data collected and the findings of the study. Reflexivity is important, as it sheds light on how the relationship between researchers and participants played a role in the study and measures taken by researchers to mitigate their biases. The authors have mentioned how they dealt with their biases, assumptions, and positions in the analysis, and their identities in the methodological rigour in the manuscript, I would suggest writing a separate section on reflexivity to briefly discuss their identities, positions, assumptions and backgrounds and how they affected the study.

Methodological rigour

The authors mentioned that the description of data collection procedures ensures transferability. Transferability in qualitative research is more about transferring the study findings to a setting with similar context and characteristics. The authors need to explain how they define transferability in this context.

The authors said that they bracketed their experiences in this study. Many scholars, including Heidegger, argue that it is almost impossible to distance from one’s self [values, experiences, beliefs etc] in research, it would be helpful to hear from the authors how they applied the concept of bracketing and how it has affected the study.

Results

The authors have well presented the study results and the interpretation of the findings, which are supported by the data. However, there is an issue concerning the selection and allocation of quotes. For example, in many areas, the authors assigned more than one quote from different participants where they talked about the experience of one participant.

Also, adding a summary concluding each theme would add value to the results.

In table 2, the authors presented the overarching theme identified in this study. However, the theme does not reflect the experience of the LGBTQ+ community specifically. I would suggest checking and re-defining this theme.

Life in the family

On p. 9, the authors said: “Another participant narrated how the mother loves….” however, they appended 2 quotes from 2 participants. I would recommend the authors revise this repeating errors throughout the results section.

• Conclusion

The authors have summarised the findings of the study, but this section does not provide the meanings and significance of the study. I would recommend revising the conclusion section to state the implications of the study on policy-making and practices in the study setting, and how this study can be used to address social disapproval and stigma experienced by the LGBTQ+ community members.

Also, I would recommend the authors direct specific recommendations to specific institutions, individuals, communities etc according to the study findings.

• Limitations of the study

Limitations of the study should be discussed before conclusions are made. I would suggest moving this section.

Reviewer #2: Overall, this study was properly conducted and well-written.

1.The introduction, aim, and method section were clearly written. Starting from the concise introduction, an explicit statement of the research aim, appropriate research design, clear description of the study area and population, appropriate sample size, data collection techniques, and data analysis.

2.The results were adequately analyzed and presented, with the major finding being the stigma the LQBTQ community undergo from the general public and their families were stated. This stigma led to this group having inadequate mental health. However, they got support from the other member of their community.

3.The discussion was well-detailed, comparing the findings with the broader literature.

4.A limitation of the study, where only a limited members of the study population (LQBTQ) was identified, was also noted. The authors highlighted the few implications to practice and policy.

5.However, no implications were stated for future research.

6.The conclusion effectively addressed the aim of the research, with the recommendations also provided.

7.The individual contribution of each of the authors were not found.

**********

6. PLOS authors have the option to publish the peer review history of their article (what does this mean? ). If published, this will include your full peer review and any attached files.

If you choose “no”, your identity will remain anonymous but your review may still be made public.

**Do you want your identity to be public for this peer review?** For information about this choice, including consent withdrawal, please see our Privacy Policy .

Reviewer #1: **Yes: ** Jean Paul Bikorimana

Reviewer #2: No

**********

[NOTE: If reviewer comments were submitted as an attachment file, they will be attached to this email and accessible via the submission site. Please log into your account, locate the manuscript record, and check for the action link "View Attachments". If this link does not appear, there are no attachment files.]

While revising your submission, please upload your figure files to the Preflight Analysis and Conversion Engine (PACE) digital diagnostic tool, https://pacev2.apexcovantage.com/ . PACE helps ensure that figures meet PLOS requirements. To use PACE, you must first register as a user. Registration is free. Then, login and navigate to the UPLOAD tab, where you will find detailed instructions on how to use the tool. If you encounter any issues or have any questions when using PACE, please email PLOS at figures@plos.org . Please note that Supporting Information files do not need this step.

Attachment Submitted filename: MAR 23 2025-Konlan.docx

# Author comment: Author response to Decision Letter 0

**DOI:** 10.1371/journal.pone.0325148.r003

21 Apr 2025

University of Ghana

College of Health Sciences

School of Nursing and Midwifery

Department of Adult Health

20th April, 2025

The Editor

PLOS ONE

Dear Sir/Madam,

RESPONSE TO REVIEWERS

We are grateful for the review of our manuscript titled “Self-reported life experiences of members of the LGBTQ+ community in Accra, Ghana” to make it better for possible publication.

Comments from the editor

Journal Requirements:

When submitting your revision, we need you to address these additional requirements.

1.Please ensure that your manuscript meets PLOS ONE's style requirements, including those for file naming. The PLOS ONE style templates can be found at

https://journals.plos.org/plosone/s/file?id=wjVg/PLOSOne_formatting_sample_main_body.pdf and

https://journals.plos.org/plosone/s/file?id=ba62/PLOSOne_formatting_sample_title_authors_affiliations.pdf

2. We note that your Data Availability Statement is currently as follows: All relevant data are within the manuscript and in Supporting Information files.

Please confirm at this time whether or not your submission contains all raw data required to replicate the results of your study. Authors must share the “minimal data set” for their submission. PLOS defines the minimal data set to consist of the data required to replicate all study findings reported in the article, as well as related metadata and methods (https://journals.plos.org/plosone/s/data-availability#loc-minimal-data-set-definition).

For example, authors should submit the following data:

- The values behind the means, standard deviations and other measures reported;

- The values used to build graphs;

- The points extracted from images for analysis.

Authors do not need to submit their entire data set if only a portion of the data was used in the reported study.

If your submission does not contain these data, please either upload them as Supporting Information files or deposit them to a stable, public repository and provide us with the relevant URLs, DOIs, or accession numbers. For a list of recommended repositories, please see https://journals.plos.org/plosone/s/recommended-repositories.

If there are ethical or legal restrictions on sharing a de-identified data set, please explain them in detail (e.g., data contain potentially sensitive information, data are owned by a third-party organization, etc.) and who has imposed them (e.g., an ethics committee). Please also provide contact information for a data access committee, ethics committee, or other institutional body to which data requests may be sent. If data are owned by a third party, please indicate how others may request data access.

3. Please include your full ethics statement in the ‘Methods’ section of your manuscript file. In your statement, please include the full name of the IRB or ethics committee who approved or waived your study, as well as whether or not you obtained informed written or verbal consent. If consent was waived for your study, please include this information in your statement as well.

4. Please review your reference list to ensure that it is complete and correct. If you have cited papers that have been retracted, please include the rationale for doing so in the manuscript text, or remove these references and replace them with relevant current references. Any changes to the reference list should be mentioned in the rebuttal letter that accompanies your revised manuscript. If you need to cite a retracted article, indicate the article’s retracted status in the References list and also include a citation and full reference for the retraction notice.

[Note: HTML markup is below. Please do not edit.]

Response to editor

1. We have complied with the requirements for file naming as suggested by the editor.

2. We have complied with the suggestions on data availaibility by providing the recommended data availability statement in the manuscript. This is on page 21 of the revised manuscript.

3. We have included the full ethics statement in the methods section of the revised manuscript . This can be found on page 9 of the revised manuscript.

4. We have revised the reference list to make it complete as suggested by the reviewer. This can be found on pages 21 to 23 of the revised manuscript

Editor’s comments

hope this message finds you well. Thank you for submitting your manuscript titled "Self-reported life experiences of members of the LGBTQ+ community in Accra, Ghana" to our journal. After careful review, we have determined that your manuscript requires minor revisions before we can proceed with further decisions. Below, I have outlined the revisions/improvements needed:

1. Abstract

• Revise the Results section:

o Replace “other geographical settings” with “within their families” to align with the identified themes.

o Alternatively, clarify what "other geographical settings" refers to.

• Revise the Conclusion section:

o Clearly indicate the target audience for the recommendations (e.g., government, policymakers, global community).

2. Introduction

• Clearly state the research gap and the specific problem being addressed.

• Introduce "life experiences" earlier in the introduction to establish its central role in the study.

• Engage with relevant literature on life experiences of LGBTQ+ individuals to justify the study’s significance.

• Connect policies and laws (macro level) to the lived experiences (micro level) of LGBTQ+ individuals.

• Ensure smooth transitions between ideas, particularly before the sentence: “There is little information in the literature on the life experiences of LGBTQ+ persons in the Ghanaian society.”

3. Methods

3.1 Study Setting

• Briefly discuss gender and sexual orientation context in Ghana to provide a deeper understanding of the setting.

3.2 Population, Sampling, and Sample Size

• Ensure all details related to participant selection and sample size are contained in this section.

• Clarify how participants were recruited, especially if they were recruited in clinics.

o Address ethical considerations related to participant recruitment, particularly concerning safety and security.

o Specify whether participants were informed beforehand and how access to them was gained.

• Move the sentence about data saturation ("Data collection from participants was done until no new information emerged.") to the Data Collection section.

3.3 Data Collection and Tools

• Revise the section title from "Selection of Participants and Data Collection" to "Data Collection and Tools."

• Indicate where the interviews were conducted and the rationale for selecting those locations to ensure a natural interview process.

3.4 Data Analysis

• Add a section on Reflexivity to discuss:

o Researcher positionality, assumptions, and biases.

o How power dynamics between researcher and participants were managed.

o Measures taken to ensure credibility of analysis.

• Clarify the concept of bracketing and how it was applied, considering scholarly critiques on its feasibility.

3.5 Methodological Rigor

• Clearly define transferability in the study’s context.

• Provide details on how bracketing was applied and its impact on the study.

4. Results

• Ensure consistent allocation of participant quotes (avoid attributing multiple quotes to a single participant).

• Add a summary at the end of each theme to synthesize key findings.

• Revise Table 2 to ensure the overarching theme directly reflects LGBTQ+ experiences.

4.1 Life in the Family

• Ensure descriptions match the corresponding quotes (e.g., revise "Another participant narrated…" when multiple quotes follow).

5. Discussion

• The discussion is strong but ensure integration of qualitative and quantitative findings where applicable.

6. Conclusion

• Revise the conclusion to:

o Clearly state the significance and implications of the study for policy and practice.

o Provide targeted recommendations for specific audiences (e.g., government, policymakers, community organizations).

7. Limitations of the Study

• Move this section before the conclusion for logical flow.

• Ensure limitations are clearly stated, including:

o The small sample size and its implications.

o The potential influence of the study setting on findings.

8. Implications for Future Research

• Add a specific section on future research to:

o Identify areas needing further study (e.g., larger-scale research, intersectionality, mental health impacts).

9. Author Contributions

• Include a statement detailing each author’s contribution to align with journal requirements.

By addressing these comments, you can improve the comprehensiveness, relevance, and clarity of your research article.

Please address these revisions within 45 days, by or before May 7, 2025. If you have any questions or need further clarification on any of the points mentioned above, please do not hesitate to contact us.

Thank you for your attention to these revisions. We look forward to receiving the updated manuscript.

Best regards,

Mc Rollyn D. Vallespin

Academic Editor

PLOS ONE

Responses of Authors to Review

1. Abstract

• We have reviewed the results section of the abstract as indicated on page 2 of the revised manuscript:

o We have edited the statement and removed “other geographical settings” as suggested by the reviewer

• Revise the Conclusion section:

o We have stated the specific persons for which the recommendations are targeted as indicated on page 2 of the revised manuscript

2. Introduction

• We have identified the gap in the literature for which this study was conducted as suggested by the reviewer. This is found on pages 3 and 4 of the revised manuscript.

• We have introduced life experiences in the earlier part of the introduction of the manuscript as suggested by the reviewer. This is found on page 3 of the revised manuscript.

• We have justified the significance of the study with relevant literature as indicated on pages 3 and 4 of the revised manuscript.

• We have ensured a transition in ideas as suggested by the reviewer in the introduction. This is found on pages 3 and 4 of the revised manuscript.

3. Methods

3.1 Study Setting

• We have provided the specific gender dynamics and sexual orientation of the study setting as indicated on pages 4 and 5 of the revised manuscript.

3.2 Population, Sampling, and Sample Size

• We have provided information on participant selection and sample size in this section. This is indicated on page 5 of the revised manuscript.

• We have clarified how the participants were recruited as well as the specific way by which the researchers gained access to the study participants. This is found on pages 6 and 7 of the revised manuscript on data collection

• We have moved the information on data saturation to the data collection section on pages 6 and 7 of the revised manuscript.

3.3 Data Collection and Tools

• We have revised the section title from "Selection of Participants and Data Collection" to "Data Collection and Tools" as suggested by the review. This can be found on page 6 of the revised manuscript.

• We have indicated where the interviews were conducted in the revised manuscript on page 6 specifically at Data collection and tool

3.4 Data Analysis

o We have included a section on reflexivity as suggested by the reviewer. This is found on page 8 of the revised manuscript. We have discussed our positionality, assumptions, and biases as well as the power dynamics between researcher and participants were managed.

o We have further discussed measures used to ensure credibility of the findings in the revised manuscript as indicated on pages 7 and 8 of the revised manuscript.

• We have discussed bracketing of our experiences under methodological rigour discussed on page 7 of the revised manuscript.

3.5 Methodological Rigor

• We have discussed the issues of bracketing in the revised manuscript on page 7.

4. Results

• We have ensured consistent allocation of participant quotes as suggested by the reviewer throughout the results section.

• We have revised Table 2 pursuant to the suggestions of the review. This can be found on page 11 of the revised manuscript.

4.1 Life in the Family

• We have revised the quotes under this section to reflect the directions of the reviewer.

5. Discussion

• We have re-written the discussions as suggested by the reviewer. This can be found on pages 17 to 20 of the revised manuscript.

6. Conclusion

• We have revised the conclusion of the study and clearly stated the implication of the findings for policy makers. This can be found on page 20 of the revised manuscript.

7. Limitations of the Study

• We have moved the limitation to now come before the conclusion as suggested by the reviewer. This can be found on page 20 of the revised manuscript.

• We have discussed specific limitations related to sampling and the setting on the findings as suggested by the reviewer. This can be found on page 20 of the revised manuscript.

8. Implications for Future Research

o We have discussed implications for future research in the limitations section of the revised manuscript on page 20.

9. Author Contributions

• We have included the contributions of the authors to the revised manuscript as suggested by the reviewer on page 21 of the revised manuscript

We hope our manuscript would be considered for publication.

Thank you

Yours sincerely,

Dr. Kennedy Dodam Konlan

(Corresponding author)

Attachment Submitted filename: Response to Reviewers.pdf

# Editor report: Decision Letter 1

**DOI:** 10.1371/journal.pone.0325148.r004

**Authors:** Daryl Higgins

7 May 2025

Self-reported life experiences of members of the LGBTQ+ community in Accra, Ghana

PONE-D-24-54894R1

Dear Dr. Konlan,

We’re pleased to inform you that your manuscript has been judged scientifically suitable for publication and will be formally accepted for publication once it meets all outstanding technical requirements.

Within one week, you’ll receive an e-mail detailing the required amendments. When these have been addressed, you’ll receive a formal acceptance letter and your manuscript will be scheduled for publication.

An invoice will be generated when your article is formally accepted. Please note, if your institution has a publishing partnership with PLOS and your article meets the relevant criteria, all or part of your publication costs will be covered. Please make sure your user information is up-to-date by logging into Editorial Manager at Editorial Manager®  and clicking the ‘Update My Information' link at the top of the page. If you have any questions relating to publication charges, please contact our Author Billing department directly at authorbilling@plos.org.

If your institution or institutions have a press office, please notify them about your upcoming paper to help maximize its impact. If they’ll be preparing press materials, please inform our press team as soon as possible -- no later than 48 hours after receiving the formal acceptance. Your manuscript will remain under strict press embargo until 2 pm Eastern Time on the date of publication. For more information, please contact onepress@plos.org.

Kind regards,

Daryl Higgins, PhD

Academic Editor

PLOS ONE

Additional Editor Comments (optional):

Please note there is a small typographical error in the Limitations section. it says: "...particularly none residents of Accra." but should be "...particularly non-residents of Accra."

Reviewers' comments:

# Editor report: Acceptance letter

**DOI:** 10.1371/journal.pone.0325148.r005

**Authors:** Daryl Higgins

PONE-D-24-54894R1

PLOS ONE

Dear Dr. Konlan,

I'm pleased to inform you that your manuscript has been deemed suitable for publication in PLOS ONE. Congratulations! Your manuscript is now being handed over to our production team.

At this stage, our production department will prepare your paper for publication. This includes ensuring the following:

* All references, tables, and figures are properly cited

* All relevant supporting information is included in the manuscript submission,

* There are no issues that prevent the paper from being properly typeset

You will receive further instructions from the production team, including instructions on how to review your proof when it is ready. Please keep in mind that we are working through a large volume of accepted articles, so please give us a few days to review your paper and let you know the next and final steps.

Lastly, if your institution or institutions have a press office, please let them know about your upcoming paper now to help maximize its impact. If they'll be preparing press materials, please inform our press team within the next 48 hours. Your manuscript will remain under strict press embargo until 2 pm Eastern Time on the date of publication. For more information, please contact onepress@plos.org.

If we can help with anything else, please email us at customercare@plos.org.

Thank you for submitting your work to PLOS ONE and supporting open access.

Kind regards,

PLOS ONE Editorial Office Staff

on behalf of

Professor Daryl Higgins

Academic Editor

PLOS ONE
